# Aberrant Synaptic PTEN in Symptomatic Alzheimer’s Patients May Link Synaptic Depression to Network Failure

**DOI:** 10.3389/fnsyn.2021.683290

**Published:** 2021-05-11

**Authors:** Marta Díaz González, Assaf Buberman, Miguel Morales, Isidro Ferrer, Shira Knafo

**Affiliations:** ^1^Department of Physiology and Cell Biology, Faculty of Health Sciences, The National Institute for Biotechnology in the Negev, Ben-Gurion University of the Negev, Beer-Sheva, Israel; ^2^Instituto Biofisika (UPV/EHU, CSIC), University of the Basque Country, Leioa, Spain; ^3^Department of Pathology and Experimental Therapeutics, Biomedical Network Research Center of Neurodegenerative Diseases (CIBERNED), Biomedical Research Institute of Bellvitge (IDIBELL), Service of Pathologic Anatomy, Bellvitge University Hospital, University of Barcelona, L’Hospitalet de Llobregat, Spain; ^4^Ikerbasque, Basque Foundation for Science, Bilbao, Spain

**Keywords:** hippocampus, plasticity, human, cognition, synaptosomes, PSD-95

## Abstract

In Alzheimer’s disease (AD), Amyloid β (Aβ) impairs synaptic function by inhibiting long-term potentiation (LTP), and by facilitating long-term depression (LTD). There is now evidence from AD models that Aβ provokes this shift toward synaptic depression by triggering the access to and accumulation of PTEN in the postsynaptic terminal of hippocampal neurons. Here we quantified the PTEN in 196,138 individual excitatory dentate gyrus synapses from AD patients at different stages of the disease and from controls with no neuropathological findings. We detected a gradual increase of synaptic PTEN in AD brains as the disease progresses, in conjunction with a significant decrease in synaptic density. The synapses that remain in symptomatic AD patients are more likely to be smaller and exhibit fewer AMPA receptors (AMPARs). Hence, a high Aβ load appears to strongly compromise human hippocampal synapses, as reflected by an increase in PTEN, inducing a loss of AMPARs that may eventually provoke synaptic failure and loss.

## Introduction

Alzheimer’s disease (AD) is the most common cause of dementia in western countries, and given the increasing longevity of many populations worldwide, the social and economic impact of AD is mounting (Frozza et al., 2018). Clinically, AD most often presents with the subtle onset of memory loss and the inability to form new memories, followed by the slow progression of dementia over the course of several years. In the brain, the dentate gyrus (DG) resists the formation of plaques, tangles, and neurodegeneration until the later stages of AD, although changes regarding its dissociation from other regions may contribute to the cognitive disturbances in AD, as might other intrinsic alterations (Ohm, 2007). Granular cells in the DG receive excitatory synaptic inputs from the medial and lateral entorhinal cortex (EC) via the perforant path (Woods et al., 2018). Inputs from the EC to the DG play a role in pattern separation, a process that involves the storage of similar experiences using non-overlapping memory (Yassa and Stark, 2011) and that is involved in the initiation of memory retrieval from the hippocampal CA3 (Lee and Kesner, 2004). Such events become disrupted in the aging brain (Yassa et al., 2011), and indeed, acute DG inactivation impairs memory encoding (Kheirbek et al., 2013; Madroñal et al., 2016) and retrieval (Tayler et al., 2013). Thus, DG networks are essential for the encoding, retrieval, and discrimination of memories (Ohm, 2007; Hainmueller and Bartos, 2020).

Because there is as yet no specific treatment available, the management of AD is difficult and frustrating with a primary focus on the long-term amelioration of associated behavioral and neurological problems (McCall, 2013; Noroozian, 2016; Cummings et al., 2018; Frozza et al., 2018; Sabbagh et al., 2019). To develop effective therapies for AD, it is necessary to fully understand the pathophysiological mechanisms that lead to cognitive impairment in the early stages of this disorder. The accumulation of Amyloid β peptide (Aβ) may initially impair cognitive function, causing mild alterations to hippocampal synaptic function (Selkoe, 2002; Palop and Mucke, 2010; Motta et al., 2018; Tanaka et al., 2019) prior to the appearance of synaptic loss and cell death (Selkoe, 2002; Cummings et al., 2015). However, the exact molecular mechanisms that link Aβ to synaptic and cognitive impairment are not fully understood, although there is now growing evidence that Aβ triggers a pathologically enhanced form of synaptic depression (Hsieh et al., 2006; Knafo et al., 2016; Knafo and Esteban, 2017).

One protein strongly linked to Aβ-dependent synaptic depression is PTEN (Phosphatase and Tensin Homolog Deleted on Chromosome Ten). PTEN participates in NMDA-dependent long-term depression (LTD) in the adult brain (Jurado et al., 2010; Knafo et al., 2016; Sanchez-Puelles et al., 2020), and it may mediate the synaptic plasticity that contributes to cognitive processes and normal brain function (Takeuchi et al., 2013; Hobert et al., 2014; Knafo et al., 2016; Knafo and Esteban, 2017; Wang et al., 2017; Sanchez-Puelles et al., 2020). We previously showed that Aβ triggers the entry of PTEN into the postsynaptic compartment, where it accumulates (Knafo et al., 2016) and can interact with PDZ proteins, possibly PSD-95 (Jurado et al., 2010; Knafo et al., 2016; Sanchez-Puelles et al., 2020). The activity of PTEN at synapses provokes a local downregulation of the PIP_3_ pathway, and eventually, it triggers the endocytosis of α-amino-3-hydroxy-5-methyl-4-isoxazole propionic acid receptors (AMPARs) that causes synaptic depression (Arendt et al., 2010; Jurado et al., 2010; Knafo et al., 2016).

This study aimed to validate the results obtained in AD models and to precisely assess the PTEN and AMPAR that can be found in hippocampal synapses. We also examined the relationship between these parameters to the Aβ load, as reflected by the intracellular and extracellular hippocampal Aβ. As such, we scanned with a super-resolution confocal microscopy preparations of the human hippocampal formation immunostained for PSD-95 as a postsynaptic marker and for PTEN. We analyzed the intensity of PTEN fluorescence within each PSD-95 puncta. Doing so in thousands of puncta in patients at different stages of AD indicated that there is significantly more PTEN within PSD-95 puncta at advanced AD stages, while the density of PSD-95 puncta diminishes. We isolated synaptosomes from human hippocampal preparations and examined the levels of the AMPAR subunit GluA1, witnessing a waning of synaptic GluA1 in advanced AD cases. Our results suggest that AD progression is associated with an increase in Aβ load and a loss of excitatory synapses. The synapses remaining are likely to be smaller, and they contain higher levels of PTEN and lower levels of GluA1-containing AMPARs. These pathological alterations to synapses probably trigger substantial changes in the connectivity between the EC and the hippocampal formation, which may produce cognitive impairment and dementia.

## Materials and Methods

All the experiments carried out in this study were performed blind and each specimen was given a code that was not broken until after the analysis had terminated.

### Patient Selection

**FIGURE 1:**
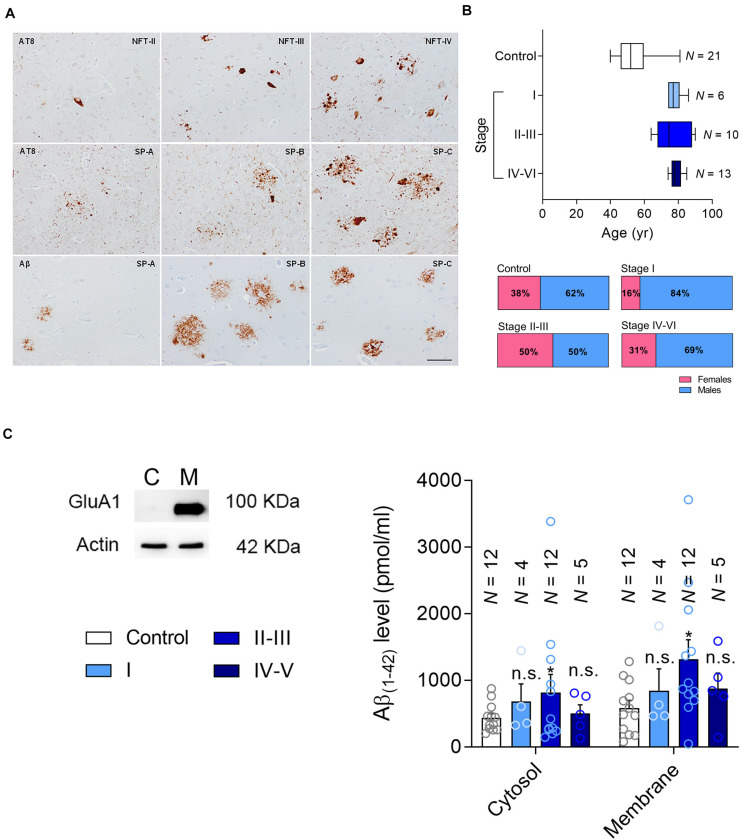
Patient classification and Aβ measurement. **(A)** Representative changes in pathological Alzheimer’s disease revealed with anti-phosphorylated tau (clone AT8) and Aβ antibodies, categorized following the Braak and Braak nomenclature adapted for immunohistochemistry in paraffin sections: NFT II, III and IV indicate neurofibrillary tangle pathology stages II, III and IV; SP A, B and C refer to senile plaque burden stages A, B and C. The upper row mainly focuses on the NFT pathology, whereas the middle row on the tau pathology surrounding amyloid deposits. The lower row shows the morphology of diffuse plaques on the left and the core plaques on the right, with a mixture of them in the central image. **(B)** The age range (Top) and gender of the patients in the current cohort (Bottom). **(C)** Left, the hippocampal formation was fractionated into the cytosolic (C) and membrane fractions (M), testing the efficacy of this separation in western blots probed for the AMPAR subunit as a marker of the membrane fraction marker. Right, Aβ levels were determined by ELISA [pg/μg protein] in the cytosolic and membrane fractions. N is the number of patients, and the data are presented as the average ± SEM.

Neuropathological categorization was performed according to the Braak and Braak classification adapted for paraffin sections (Braak et al., 2006) (representative examples of sections for each of the stages are shown in Figure 1). The samples from patients were classified as controls (showing no neuropathological findings or lesions), asymptomatic AD (Braak and Braak stage I–III) or symptomatic AD (Braak and Braak stage IV–VI). Please refer to Table 1 for the patient details and to Supplementary Table 1 for details on the average age in each experiment.

**TABLE 1:** Patients classification, age, and post-mortem interval.

**Classification**	**Age (y)**	**Gender**	**Post-mortem interval (h)**	**Braak and braak stage**
Control	65	F	4	No pathological findings
Control	63	M	17	No pathological findings
Control	46	F	9	No pathological findings
Control	43	M	5	No pathological findings
Control	46	M	15	No pathological findings
Control	43	M	4	No pathological findings
Control	52	M	3	No pathological findings
Control	50	M	17	No pathological findings
Control	51	F	4	No pathological findings
Control	53	M	3	No pathological findings
Control	46	F	14	No pathological findings
Control	52	M	3	No pathological findings
Control	47	M	10	No pathological findings
Control	55	M	13	No pathological findings
Control	55	M	5	No pathological findings
Control	65	F	5	No pathological findings
Control	81	F	4	No pathological findings
Control	66	F	4	No pathological findings
Control	48	F	4	No pathological findings
Control	56	M	4	No pathological findings
Control	40	M	5	No pathological findings
Asymptomatic AD	78	M	7	Stage I
Asymptomatic AD	86	M	18	Stage I
Asymptomatic AD	79	M	5	Stage I
Asymptomatic AD	74	M	6	Stage I
Asymptomatic AD	76	M	4	Stage I
Asymptomatic AD	74	F	2	Stage I
Asymptomatic AD	72	M	8	Stage II
Asymptomatic AD	71	M	5	Stage II
Asymptomatic AD	67	M	7	Stage II
Asymptomatic AD	88	F	5	Stage II
Asymptomatic AD	83	M	18	Stage III
Asymptomatic AD	88	F	1	Stage III
Asymptomatic AD	64	M	4	Stage III
Asymptomatic AD	68	F	5	Stage III
Asymptomatic AD	77	F	3	Stage III
Asymptomatic AD	90	M	2	Stage III
Symptomatic AD	80	F	2	Stage IV
Symptomatic AD	82	M	5	Stage IV
Symptomatic AD	84	M	12	Stage IV
Symptomatic AD	74	M	4	Stage IV
Symptomatic AD	75	M	6	Stage IV
Symptomatic AD	77	M	19	Stage IV
Symptomatic AD	81	M	3	Stage IV
Symptomatic AD	79	M	4	Stage IV
Symptomatic AD	78	M	16	Stage V
Symptomatic AD	78	M	9	Stage V
Symptomatic AD	85	F	16	Stage V
Symptomatic AD	74	F	5	Stage V
Symptomatic AD	77	F	3	Stage VI

### Tissue Preparation

The whole hippocampus was collected at autopsy and cut into 7–12 mm thick blocks transverse to the long axis, maintaining them at -80°C until use. Subsequently, series of cryostat sections (4 μm) were obtained at –20°C (Leica CM3050s, Leica Biosystems) and mounted on frozen gelatinized slides. The tissue was fixed in 4% paraformaldehyde for 10 min at 4°C and processed for immunohistochemistry. This procedure allowed 10–12 different specimens to be processed in parallel. Staining for phosphorylated tau (clone AT8) and β-amyloid (Aβ) were performed on one series, DAPI staining was combined with PSD-95 and PTEN on the next consecutive sections, and DAPI staining combined with Thioflavin-S was performed on the following consecutive sections.

### Braak and Braak Classification

Paraffin sections were processed for immunohistochemistry using the AT8 (Innogenetics, Ghent, BE) and Aβ (Dako, Glostrup, DK) antibodies, both at a dilution of 1:50, the binding of which was detected with EnVision+ System peroxidase (Dako, Agilent Technologies, Barcelona, Spain). The immunoreaction was visualized with diaminobenzidine and H_2_O_2_, and the sections were counterstained lightly with haematoxylin (bar = 50 μm). The patients’ AD pathology was categorized using the Braak and Braak scale adapted to immunohistochemistry on paraffin sections.

### ELISA

We first verified that the cytosolic/membrane fraction had been obtained correctly in western blots (Figure 1C), analyzing the Aβ42 monomers with the Human Aβ42 ELISA Kit (Thermofisher Scientific). The standard curve of the Aβ42 peptides was obtained with *serial* dilutions of the standard peptides. The standard peptide was included in the kit, and it was diluted in “Reconstitution Buffer” (also included in the kit) according to the volume marked on the label. For Aβ quantification, samples were diluted with standard dilution buffer (supplied with the kit) to keep the concentration within the detectable range (1–100 pg/ml). After the addition of the stop solution, the 96-well plate was read at 450 nm, and the sample concentration was calculated from a standard curve prepared using purified Aβ of various concentrations.

### Thioflavin-S Staining

After fixing, the sections were dehydrated through a series of ethyl alcohol solutions (70, 80, 95, and 100%, 2 min in each) and then stained with 1% Thioflavin S for 30–60 min. The sections were then rehydrated and dipped in DAPI. After staining, the sections were covered with ProLong Gold (Thermo Fisher Scientific) and coverslipped.

### Immunofluorescence

Fixed sections were permeabilized with 0.1% Triton X-100 for 30 min and non-specific antibody binding was avoided by incubating at room temperature (RT) for 2 h in blocking solution (5% horse serum, 0.1% Triton X-100 in PBS). Dual immunohistochemistry was then performed sequentially to study PSD-95 (1:250 in blocking solution: D74D3, Cell Signaling Technology) and PTEN (1:200 in blocking solution: AMB-2052, Cascade BioSciences) using primary monoclonal antibodies. Antibody binding was detected for 30 min at 37°C with their corresponding secondary antibodies: donkey anti-mouse conjugated to Alexa Fluor 350 (1:1000 in blocking solution: Invitrogen) and goat anti-rabbit conjugated to Alexa Fluor 594 (1:1000 in blocking solution: Life Technologies). After staining, the sections were mounted with ProLong Gold (Thermo Fisher Scientific) and coverslipped. All the samples were processed in parallel.

### Imaging and Quantification

#### Aβ Plaques

**FIGURE 2:**
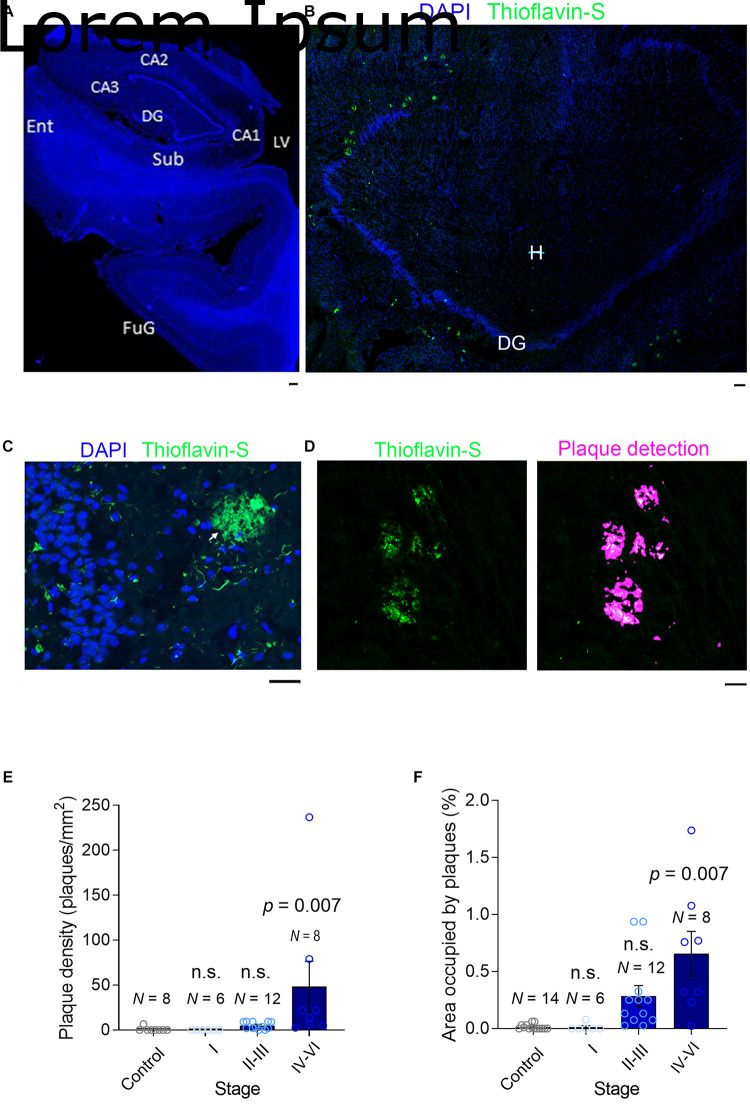
Plaque detection in AD samples. **(A)** A slide scanner image (×5) of a DAPI-stained section (stage II) containing the hippocampal formation: Sub, Subiculum; Ent, Entorhinal Cortex; DG, Dentate Gyrus; LV, Lateral Ventricle; CA1-3, Cornu Ammonis; FuG, Fusiform Gyrus. **(B)** A confocal image (×10) of a section (stage V) containing mainly the dentate gyrus stained with DAPI (blue) and Thioflavin-S to detect the Aβ plaques (green): DG, Dentate Gyrus; H, Hilus. **(C)** High magnification image (×40) of the dentate gyrus (stage V) where small Aβ aggregates can be seen in the granule cell layer (GCL, green) and an extensive Aβ plaque is seen in the molecular layer (ML). **(D)** Left, Part of a section scanned with a slide scanner (×10) with Aβ plaques. Right, automatic detection of plaques, used to calculate the plaque density and the area occupied by plaques. **(E,F)** A bar graph showing the plaque density and the area occupied by plaque. N is the number of patients, and the data are presented as the average ± SEM. The scale bar in all the images is 50 μm.

Entire DAPI/Thioflavin S-stained sections were scanned using a 3D Scan Pannoramic Histech scanner (3D Histech Kft. Budapest, Hungary) at a resolution of 0.23 μm per pixel. Images were analyzed using Pannoramic Viewer 1.15.2 SP2 RTM (3D Histech kft.) or Histoquant (3D Histech kft.) software, which provides a detailed morphometric analysis with precise measurements of different histological parameters at high resolution (Figure 2). Plaques were detected automatically, and the number of plaques per section and the area of each plaque was evaluated.

#### PSD-95 and PTEN

Super-resolution images of immunostained sections were obtained by laser-scanning confocal microscopy (Zeiss LSM 880, Carl Zeiss) over 10–20 fields in the molecular layer of the DG using identical acquisition parameters for all the sections. Images were acquired on a Zeiss LSM 880 Airyscan through an oil immersion 63× Plan-Apochromat objective (N.A.: 1.4) with a final spatial resolution of 0.075 microns/pixel. Laser excitation wavelengths were 405, 488, and 561 nm, and fluorescent emission was collected in all cases through a 1 Airy unit pinhole using selective 410–495 nm, 495–585 nm, and 585–733 nm spectra windows, respectively. Subsequently, 16 bit images were generated with a maximal sampling size according to the configuration of the optics. Quantification was performed with Imaris software (version 9.6, Bitplane AG), building three channels for PSD-95, PTEN, and DAPI. PSD-95 puncta (single dots) were identified with the built-in spot detection algorithm in Imaris, considering spots of different sizes and using local background subtraction. To follow a rigorous method for all samples and to avoid possible artifacts in the quantification, the average diameter of the spot was established as 0.3 μm for the entire process and the same threshold levels were used for all the sections. The algorithm yielded the sum of the PTEN fluorescence intensity in each PSD95 spots, as well as the individual spot sizes.

### Tissue Fractionation Into Cytosolic and Membrane Fractions

The tissue was placed in TBS [140 mM NaCl, 3 mM KCl, 25 mM Tris-HCl (PH 4.7), and 5 mM EDTA] containing a cocktail of proteases to avoid protein degradation and homogenized in a Dounce Homogenizer. Subsequently, the homogenate at 50,000 rpm was centrifuged for 1 h at 4°C (Coulter Optima L-90K Ultracentrifugue, Beckman), separating the sample into two fractions: the supernatant containing the fraction of soluble proteins; and the pellet. The pellet was resuspended in 2% SDS and centrifuged again at 4°C for 30 min at 14,000 *g* (Sorvall^TM^ Legend^TM^ Micro 21R Microcentrifuge, ThermoScientific). The supernatant from this latter centrifugation corresponds to the fraction of membrane associated proteins.

### Synaptosomal Preparation

We homogenized human hippocampal tissue in buffer A (0.32 mM sucrose, 1 mM MgCl_2_, 0.5 mM CaCl_2_, 1 mM NaHCO_3_, protease inhibitors) and centrifuged it at 1,400 *g* for 10 min to obtain the supernatant (S1) and pellet (P1). P1 was then homogenized in buffer A and centrifuged at 700 *g* for 10 min, combining the resulting supernatant with S1 and centrifuging these again at 13,800 *g* for 10 min. The supernatant obtained (S2) was further centrifuged at 100,000 *g* for 1 h and the resulting supernatant (S3) constituted the cytosolic fraction. The resulting pellet was resuspended in buffer B (0.32 mM sucrose, 1 mM NaHCO_3_, 1 mM EGTA, 1 mM dithiothreitol, protease inhibitors) and it represents the crude synaptosomal fraction used for analysis.

### Western Blotting

The lysates were collected and centrifuged at 13,000 rpm for 5 min at 4°C, and the protein in the supernatants was quantified using the BCA protein assay (Pierce, 23227) and compared to bovine serum albumin (BSA) standards. Equal amounts of total protein was prepared in 4× sample loading buffer and boiled at 95°C for 5 min immediately before electrophoresis. Proteins (10 μg per lane) were separated according to their molecular weight by SDS-PAGE in a Mini PROTEAN Tetra Cell Vertical Electrophoresis system (Bio-Rad) and in 1× running buffer [25 mM Tris (pH 8.3), 192 mM glycine, 0.1% SDS in dH_2_O]. Following electrophoresis, the proteins were transferred to a PVDF membrane (pore size 0.45 μm: Amersham Hybond, 10600023) in the Mini Trans-Blot Cell transfer system (Bio-Rad) and in 1× transfer buffer [25 mM Tris (pH 7.6), 192 mM glycine, 20% methanol in dH_2_O] for 90 min at 400 mA. The membrane was then stained with Ponceau S (Sigma Aldrich, P3504) to confirm the successful transfer of the proteins. Non-specific binding was blocked by incubation with 5% milk in TBS-T for 1 h at RT, and after washing the membranes, they were incubated for 24 h at 4°C with an antibodies against GluA1 (AMPAR subunit) (1:1,000, Rabbit: ab31232, Abcam) or PTEN (1:1,000, Rabbit, Cell Signaling Technology 9559L). The membranes were then incubated with the secondary antibodies for 1 h at RT (1:2000, HRP-conjugated anti-rabbit IgG: #7074; Cell Signaling Technology) and the immunocomplexes were visualized with a chemiluminescent agent (Immobilion^®^ Forte Western HRP Substrate, Milipore). The anti-actin antibody (1: 1,000, Rabbit Cell Signaling Technology, 4970S) was used as a protein loading control. The membranes were again washed as described above and the antibodies bound to their target proteins were detected by enhanced chemiluminescence (Luminata Forte Western HRP Substrate, Millipore, MIWBLUF0100) in a MyECL Imager (Thermo Fisher Scientific), quantifying them by densitometry using the Quantity One software (Bio-Rad).

## Results

Here we studied post-mortem hippocampal tissue from a total of 50 patients that were classified according to the Braak and Braak scale, staining brain sections with antibodies against phosphorylated tau (p-tau) and Aβ (Figure 1A). According to their clinical records and pathological classification, 16 patients had mild-to-moderate AD pathology but with no sign of cognitive impairment (asymptomatic, Braak and Braak stages I–III: further divided into stage I and stage II/III), and 13 had a more advanced pathology involving AD-related cognitive impairment (symptomatic, Braak and Braak stage IV–V). In addition, tissue from 21 younger healthy individuals was used as a control (No pathological findings). The age and gender distribution was similar for the AD patients of different pathology states (Figure 1B), and the range of the post-mortem intervals was also similar among the groups (Table 1).

### The Hippocampal Aβ Load Changes With AD Stage

The recruitment of PTEN to the postsynaptic compartment in rodent primary neurons is mediated by the Aβ (Knafo et al., 2016) generated through the proteolytic processing of the membrane-bound amyloid precursor protein (APP) (Bayer et al., 2001). Intracellular Aβ interacts with the membrane to provoke synaptic dysfunction, or alternatively, it is secreted into the extracellular space (LaFerla et al., 2007). It has been previously reported that Aβ triggers the entry of PTEN to the postsynaptic compartment (Knafo et al., 2016). To characterize the Aβ load in the hippocampal tissue, and the forms of Aβ appearing in each stage of AD, we first separated the samples into cytosolic and the membrane-bound fractions (Figure 1C), quantifying the Aβ_(1__–__42)_ monomers in each by ELISA. We found more cytosolic and membrane Aβ_(1__–__42)_ in tissue from patients at stages II–III (late asymptomatic AD) than in that from the controls. However, these values did not increase significantly in the tissue from patients at later stages of dementia, stages IV–VI (Figure 1C).

The intracellular accumulation of Aβ may lead to neuronal death and lysis, releasing Aβ into the extracellular space (LaFerla et al., 1997; D’andrea et al., 2001) where it can accumulate and oligomerize to ultimately form plaques (LaFerla et al., 1997; D’andrea et al., 2001). To quantify the Aβ aggregated into plaques, we stained fixed hippocampal slices with Thioflavin-S and scanned the entire sections at high resolution with a slide scanner (Figures 2A–C). After controlled automatic delineation of the Aβ plaque borders (Figure 2D), we quantified the plaque density and the proportion of the area occupied by plaques in each of the sections, witnessing an increase in the plaque density as the disease progressed (Figure 2E). As expected, plaques occupied a negligible proportion of the neuropil in healthy controls, as well as at early stages of AD clinically defined as asymptomatic (stages I–III). However, the proportion of plaques increased significantly as the disease advanced (Figure 2F), although even in the sections from advanced-stage patients (IV–VI, symptomatic AD), the plaques only occupied a maximum of 1.7% of the hippocampal neuropil (Figure 2F). This finding is consistent with data from transgenic mice bearing two familial AD mutations (APP/PS1) in which plaques occupy a maximum of 5% of the hippocampal neuropil (Knafo et al., 2009a,b; Merino-Serrais et al., 2011). Hence, while soluble Aβ reaches maximal values in late asymptomatic AD stages, the Aβ in plaques accumulates maximally at late symptomatic AD stages.

### High PTEN Levels in Symptomatic AD Synapses

**FIGURE 3:**
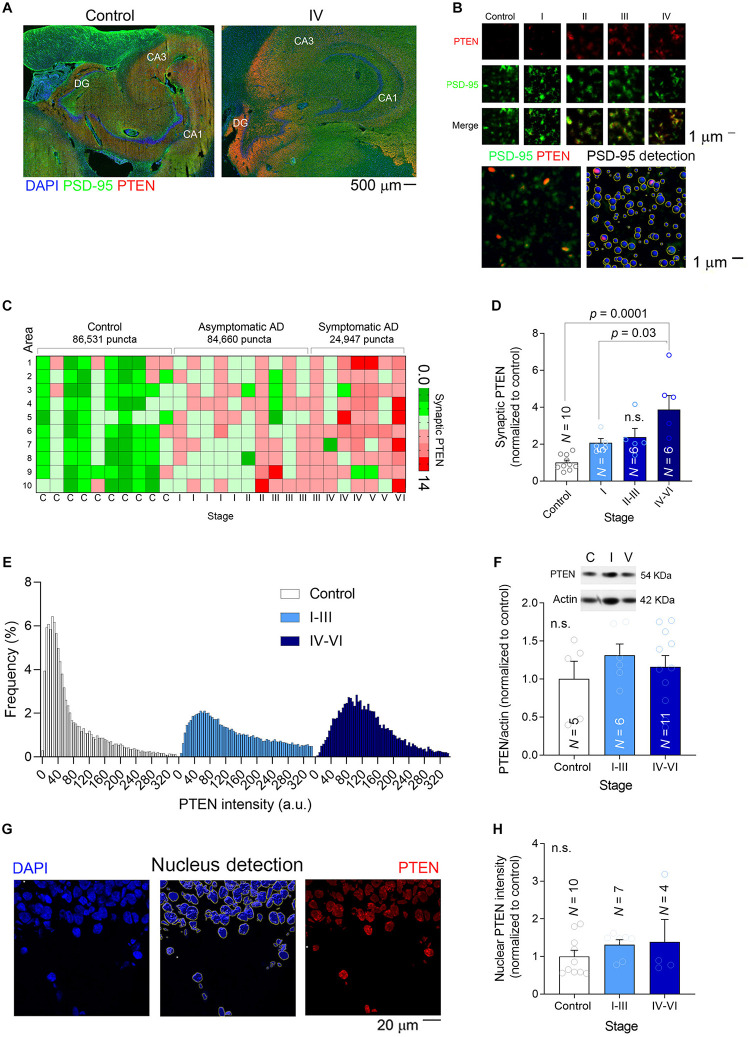
Synaptic PTEN levels are enhanced in AD patients. **(A)** Tile-scanned confocal images (×10) of the hippocampal formation stained for PTEN (red) and PSD-95 (green): DG, Dentate Gyrus; CA1-3, Cornu Ammonis. **(B)** Top, High magnification super-resolution images (63×) showing PSD-95 puncta (green) and PTEN (red) at different stages. Bottom, Detection of PSD-95 puncta with Imaris software. **(C)** Heat map showing the average relative PTEN levels (normalized to controls) in 10 high-resolution confocal fields per patient. **(D)** A bar graph depicting the relative synaptic PTEN in each group of patients. N is the number of patients, and the *P*-values were determined by One Way ANOVA followed by Tukey’s multiple comparisons test. **(E)** Frequency distribution of the PTEN in individual PSD-95 puncta. **(F)** The total amount of PTEN in brain lysates was determined in western blots. Inset, a representative example of a blot at different stages. N is the number of patients and the *P*-values were determined by One Way ANOVA. **(G)** Left, A confocal image (×40) of part of the dentate gyrus showing the nuclei stained with DAPI. Middle, The detection of nuclei to determine the amount of nuclear PTEN. Right, The pixels outside the nuclear areas were set to zero to observe only nuclear PTEN (red). **(H)** A bar graph depicting the relative nuclear PTEN in each group of patients. N is the number of patients and the *P*-value was determined by One Way ANOVA.

When recombinant PTEN expressed by dissociated hippocampal neurons is exposed to high concentrations of synthetic Aβ_(1__–__42)_ it is immediately trafficked to the postsynaptic compartment (Knafo et al., 2016). Upon its recruitment to spines, PTEN interacts with PSD-95 and it induces the endocytosis of AMAPRs, resulting in synaptic depression (Jurado et al., 2010). To determine if the hippocampal synapses of AD patients undergo such changes, we quantified the PTEN in individual human excitatory synapses in the molecular layer of the DG (Figures 3A,B). Hippocampal sections were subjected to dual immunofluorescence for PTEN and PSD-95, the latter not only interacting directly with PTEN but also serving as a marker of excitatory synapses (Figures 3A,B). After scanning the sections at high magnification (×63) with a super-resolution confocal microscope (10–20 areas per patient), we used Imaris software to detect approximately 200,000 individual PSD-95 puncta (Figure 3B). We extracted the PTEN intensity within each puncta as a measure of the amount of PTEN within the postsynaptic compartment, and crucially, there was a gradual increase in synaptic PTEN as AD pathology advanced (Figures 3C,D). A frequency distribution analysis showed that PSD-95 puncta from patients with an advanced AD pathology were more likely to contain higher levels of PTEN and less likely to have lower levels of PTEN than control puncta (Figure 3E).

Nonetheless, when the overall amount of PTEN in each hippocampal preparation was measured in western blots, significant differences could not be detected among the groups (Figure 3F). Similarly, when the PTEN fluorescence intensity was measured in individual DG nuclei (a total of 940 nuclei) we again failed to detect changes related to AD (Figures 3G,H), suggesting that the increase in PTEN levels is specific to synapses. These findings confirmed previous observations in cell models of AD in which a high Aβ load was linked to the specific recruitment of PTEN to the postsynaptic compartment (Knafo et al., 2016).

### AD Synapses Contain Fewer GluA1 Subunit-Containing AMPARs

**FIGURE 4:**
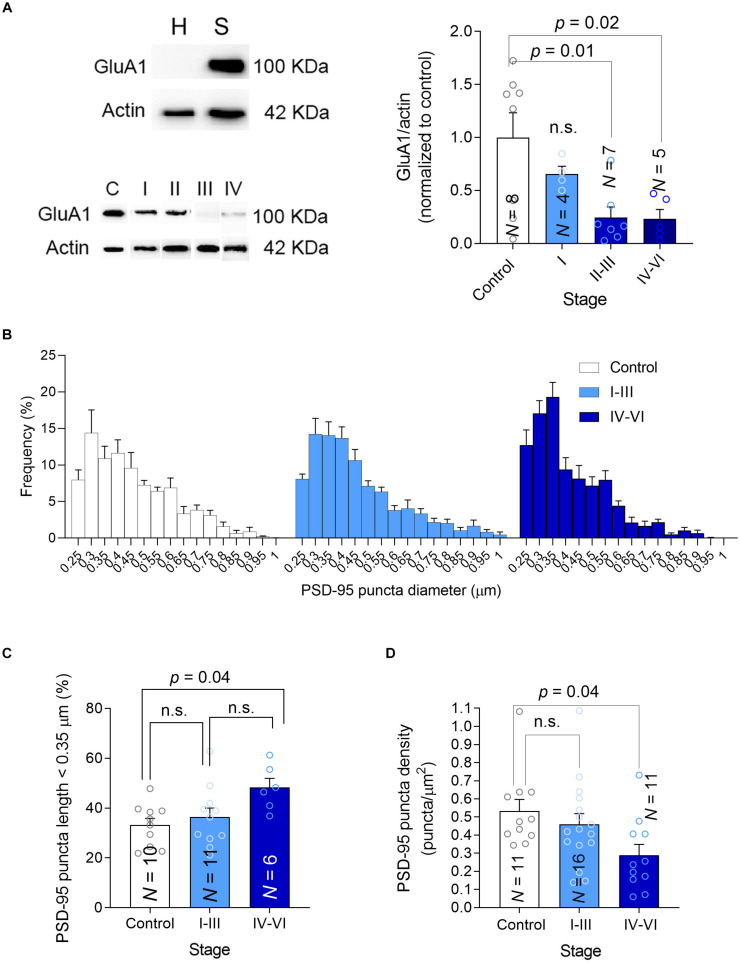
Synaptic GluA1, size and density. **(A)** Left-top, Hippocampal homogenates (H) were fractionated into crude synaptosomes (S), and the efficacy of fractionation was tested in western blots probed for the GluA1 AMPAR subunit as a synaptic marker. Left-bottom, A western blot showing the PTEN in synaptosomes, using actin as a loading control. Right, The average GluA1 in synaptosomes was determined in western blots. **(B)** Frequency size distribution of the PSD-95 puncta. **(C)** Bar graph showing the proportion (%) of small PSD-95 in each group. **(D)** Bar graph showing the density of PSD-95 puncta. In all the graphs, N is the number of patients and the data are presented as average ± SEM. *P*-values were determined by One Way ANOVA followed by Tukey’s multiple comparisons test.

A possible link between PTEN activity in synapses and cognitive deterioration in AD is the ability of PTEN to modulate the amount of AMPARs in the synapse. Indeed, PTEN regulates the number of synaptic AMPARs (Yang et al., 2014) through the PIP_3_ pathway (Jurado et al., 2010; Knafo et al., 2016; Sanchez-Puelles et al., 2020), which controls AMPAR trafficking (Qin et al., 2005) and synaptic localization (Arendt et al., 2010) in hippocampal neurons. AMPARs mediate excitatory transmission and maintain cognitive function (Knafo et al., 2012). Overexpression of PTEN leads to both cognitive impairment, impaired basal synaptic transmission, and deficits in LTP (Long Term Potentiation) (Sanchez-Puelles et al., 2020). Apart from delivering PTEN to synapses, Aβ also triggers AMPAR endocytosis and facilitates LTD (Chang et al., 2006; Hsieh et al., 2006; Gu et al., 2009; Spires-Jones and Knafo, 2012; Knafo et al., 2016; Reinders et al., 2016). Therefore, we hypothesized that synapses affected by AD pathology would contain fewer AMPARs and we tested this hypothesis in crude synaptosomal preparations isolated from human hippocampal tissue, quantifying the GluA1 synaptic AMPAR subunit in western blots. We first confirmed that our synaptosomal fractionation contained a significantly higher level of GluA1 compared with the initial lysate, implying a suitable fractionation (Figure 4A, top). We observed an apparent reduction in synaptic GluA1 that decreased gradually as the disease progressed (Figure 4A, bottom and Supplementary Figure 1). Studies on synaptic plasticity have stimulated ideas of an essential overlap between molecular pathways involved in LTD and synaptic shrinkage and/or pruning (Zhou et al., 2004; Shinoda et al., 2010; Piochon et al., 2016). To determine whether the size and the density of hippocampal synapses are affected in AD patients, we quantified the density and size of PSD-95 puncta in our preparations using Imaris software. When the frequency distributions of PSD-95 puncta diameter were analyzed there was an apparent increase in small puncta frequency (<0.35 μm: Figures 4B,C) but crucially, a reduction in PSD-95 puncta density was observed in patients with advanced symptomatic AD that suggested they had a lower synaptic density (Figure 4D). Together, these findings suggest there are fewer synapses in the hippocampus of advanced AD patients, which are more likely to be smaller and contain fewer AMPARs.

## Discussion

Understanding the molecular pathways through which the various pathological alterations in AD compromise synaptic function and potentially cause the clinical symptoms has been a long-standing goal in AD research. Testing such pathways in mouse and cell models that mimic various aspects of this pathology aids this effort. As such, we now know from cell and animal models that PTEN activity is required for NMDAR-dependent LTD and that Aβ facilitates this type of plasticity (Wang et al., 2006; Jurado et al., 2010; Sperow et al., 2012; Knafo et al., 2016; Knafo and Esteban, 2017; Sanchez-Puelles et al., 2020), establishing a potential link between PTEN and AD. One possible pathological mechanism in AD starts with Aβ overload, which leads to aberrant PTEN recruitment to spines and affecting its interactions with the postsynaptic scaffolding molecule PSD-95 in synapses, eventually triggering the endocytosis of AMPARs. These new insights indicate that this PTEN/PSD-95 interaction may represent a new candidate target for the treatment of cognitive impairment in AD. Indeed, a peptide has been designed based on PTEN’s C-terminal sequence that can block this PTEN/PSD-95 interaction and it has been successfully tested in AD model mice (Knafo et al., 2016). Subsequent studies showed that derivatives of the original peptide are stable in plasma and they are capable of crossing the blood-brain barrier *in vitro* (Lalatsa et al., 2020). Nevertheless, before continuing to develop therapies based on overcoming this pathological mechanism, it is imperative to detect parallel pathological alterations in diseased human brains.

**FIGURE 5:**
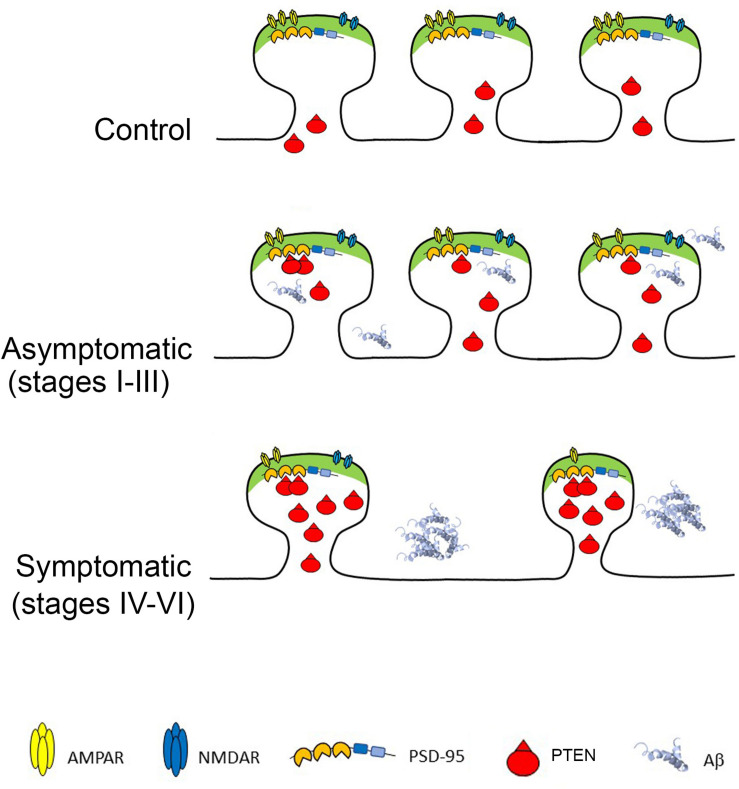
Phosphatase and Tensin Homolog Deleted on Chromosome Ten (PTEN) at AD Synapses provokes Long-Term Depression and synaptic loss. Top: Healthy synapses exhibit basal synaptic transmission due to weak PTEN activity. Middle: in response to Aβ, PTEN is recruited to the postsynaptic membrane via PDZ-dependent interactions with PSD-95. The turnover of PIP_3_ by PTEN facilitates AMPAR endocytosis and removal from synapses. Bottom: When Aβ is elevated, the sustained recruitment of PTEN at the postsynaptic membrane leads to excessive removal of AMPARs, skewing synaptic plasticity toward depression and producing chronic synaptic weakening, shrinkage and loss. The cell death provoked by Aβ overload also contributes to synaptic loss.

Here we examined the levels of PTEN in a relatively large number of individual excitatory hippocampal synapses from AD patients at different stages of the disease and from healthy controls. We found that AD hippocampal synapses at advanced, symptomatic stages contain more PTEN, which is associated with a lower density of synapses that are both smaller and have fewer GluA1-containing AMPARs. These pathological changes occur in parallel to the accumulation of Aβ first intracellularly and then extracellularly in plaques. Previous studies in cellular AD models suggest a clear cause-effect relationship between Aβ accumulation and PTEN recruitment to spines, and they confirm that blocking PTEN activity, either by inhibiting its catalytic activity or by preventing its interaction with PDZ proteins at synapses (e.g., PSD-95) can prevent the exaggerated synaptic depression and cognitive malfunctions typical of AD models. Our finding regarding the higher proportion of smaller PSD95 puncta follows a recent EM study on the hippocampal CA1 region that used a three-dimensional analysis of synapses and showed that AD synapses are slightly smaller than controls (Montero-Crespo et al., 2021). Other studies in the hippocampus (dentate gyrus and CA1) of transgenic mice revealed an increase in the proportion of small spines or decreased proportion of large spines (Knafo et al., 2009a; Merino-Serrais et al., 2011), in accordance with the current results. Combining these findings with the present study on human AD hippocampal preparations, we now propose a model describing the cascade of events occurring during AD disease progression (Figure 5).

According to this model, healthy synapses are exposed to negligible levels of Aβ and thus, PTEN mainly concentrates outside the synapses. When Aβ starts to accumulate intracellularly (some of it bound to cell membranes) it triggers the entry of PTEN into spines. This entry of PTEN requires its interaction with domains 1–2 of PSD-95 through its C-terminal PDZ motif (Jurado et al., 2010), (Knafo et al., 2016). The engagement of PTEN during Aβ overload leads to its anchoring at the postsynaptic density of dendritic spines (Jurado et al., 2010) and although the mechanism regulating this PDZ interaction remains unknown, it appears to require NMDAR activation. This enhancement of PTEN lipid phosphatase activity within the spines drives AMPR endocytosis, thereby depressing AMPAR-mediated synaptic responses, and the resulting LTD leads to synaptic shrinkage and pruning. The neurotoxicity provoked by intracellular Aβ induces cell lysis, further enhancing synaptic loss. At advanced AD stages, the hippocampus contains numerous plaques, and it may suffer largescale neurodegeneration. The neurons that remain contain fewer and weaker synapses, with elevated levels of PTEN, which may continue to compromise the synapses.

It is important to note that AD is a complex disease with multiple molecular events occurring in parallel. Thus, our model may reflect only one of the pathways that can provoke cognitive impairment and dementia, and other pathways may be altered in different patients, producing similar devastating outcomes. Nevertheless, our findings suggest that synaptic failure in AD is induced by an excess of PTEN, which shapes and reorganizes neural circuits in the hippocampal formation, making cognitive activity in the brain sub-optimal.

## Data Availability Statement

The raw data supporting the conclusions of this article will be made available by the authors, without undue reservation.

## Ethics Statement

The studies involving human participants were reviewed and approved by Institutional Review Board Statement: Brain tissue was obtained from the Institute of Neuropathology HUB-ICO-IDIBELL Biobank following Spanish legislation (Real Decreto de Biobancos 1716/2011). Informed Consent Statement: The research was conducted in compliance with the policies and principles contained in the European Policy for the Protection of Human Subjects. Tissue collection and the tracking of medical records was carried out in strict confidentiality and following local ethics committee’s protocols. All the research was conducted in compliance with the policies and principles contained in the European Policy for the Protection of Human Subjects. The patients/participants provided their written informed consent to participate in this study.

## Author Contributions

SK: conceptualization, original draft preparation, and funding acquisition. SK and MM: methodology, validation, and supervision. MD and AB: formal analysis, data curation. MD and MM: investigation. IF: resources and classification of cases. MM: project administration. All authors have read and agreed to the published version of the manuscript.

## Conflict of Interest

The authors declare that the research was conducted in the absence of any commercial or financial relationships that could be construed as a potential conflict of interest.
