# Supplementary material for: Aberrant Synaptic PTEN in Symptomatic Alzheimer’s Patients May Link Synaptic Depression to Network Failure
Source: Front Synaptic Neurosci. 2021 May 11;13:683290. doi: 10.3389/fnsyn.2021.683290 (PMC8144462; doi:10.3389/fnsyn.2021.683290)
Supplement: Supplementary file 1 [file Table_1.DOCX]

| **Suppl. Table 1: Average age of individuals in each figure.** | | | | |  |
| --- | --- | --- | --- | --- | --- |
|  |  |  |  |  |  |
|  |  | Control | I | II-III | IV |
| Fig 1c | Mean | 50.83 | 79.25 | 77.5 | 78.6 |
|  | Std. Error of Mean | 2.041 | 2.496 | 2.595 | 0.6782 |
| Fig 2e-f | Mean | 55.5 | 79.25 | 77.5 | 78.38 |
|  | Std. Error of Mean | 2.515 | 2.496 | 2.595 | 1.224 |
| Fig 3d | Mean | 51.5 | 77.83 | 78 | 78 |
|  | Std. Error of Mean | 1.778 | 1.833 | 4.171 | 0.8165 |
| Fig 4a | Mean | 51 | 79.25 | 76.14 | 78.6 |
|  | Std. Error of Mean | 3.047 | 2.496 | 3.789 | 0.6782 |
|  |  |  |  |  |  |
|  |  |  |  |  |  |
|  |  | Control | I-III | IV-VI |  |
| Fig 3f | Mean | 51.2 | 77.83 | 78 |  |
|  | Std. Error of Mean | 1.96 | 1.833 | 2.22 |  |
| Fig 3h | Mean | 51.5 | 76.5 | 80.75 |  |
|  | Std. Error of Mean | 1.778 | 1.813 | 5.706 |  |
| Fig 4c | Mean | 51.5 | 79.18 | 80.17 |  |
|  | Std. Error of Mean | 1.778 | 1.934 | 3.664 |  |
| Fig 4d | Mean | 50.45 | 77.19 | 79.36 |  |
|  | Std. Error of Mean | 1.918 | 2.003 | 1.047 |  |
