# Supplementary figures and images for: Aberrant Synaptic PTEN in Symptomatic Alzheimer’s Patients May Link Synaptic Depression to Network Failure
Source: Front Synaptic Neurosci. 2021 May 11;13:683290. doi: 10.3389/fnsyn.2021.683290 (PMC8144462; doi:10.3389/fnsyn.2021.683290)

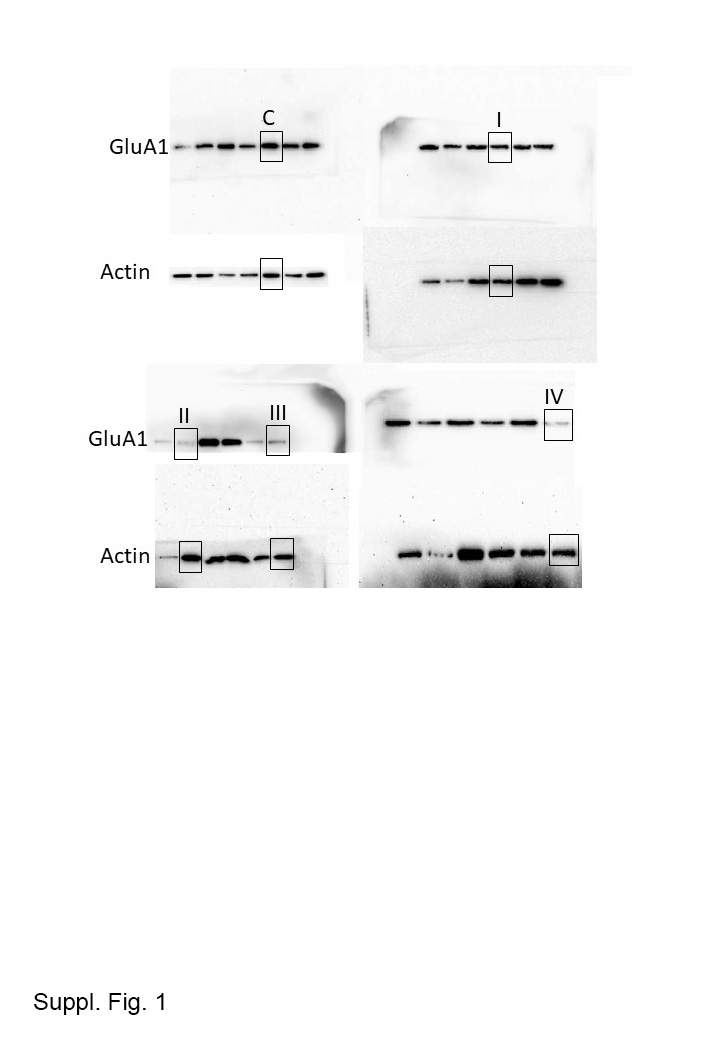

Supplement: Supplementary file 2 [file Image_1.JPEG]
